# Training to Serve: CoMACS and the Application of Ignatian Pedagogy in the Training of Healthcare Professionals in Spain

**DOI:** 10.1007/s10943-026-02575-9

**Published:** 2026-02-06

**Authors:** Ane Gutierrez-Aguirregabiria, Susana Romero-Yesa, José Ramón Sánchez-Isla

**Affiliations:** 1 Department of Medicine, Faculty of Health Sciences, University of Deusto, Bilbao, Spain; 2 Educational Innovation Unit, Faculty of Engineering, University of Deusto, Bilbao, Spain; 3 Department of Nursing, Faculty of Health Sciences, University of Deusto, Bilbao, Spain

**Keywords:** Ignatian Pedagogy, Transversal learning outcomes, Transversal competencies, Transversal skills, Learning methodologies

## Abstract

The University of Deusto, a Jesuit institution with a long educational tradition, bases its pedagogical model on Ignatian Pedagogy, promoting the comprehensive education of students. Within this framework, transversal learning outcomes occupy a central place, with the hallmark skill being that which develops self-knowledge, ethics, social responsibility, sustainability, and openness to transcendence. This approach seeks to train students who are committed to society and capable of acting with professional excellence and a sense of social justice. In this context, the Faculty of Health Sciences was created in 2020 with the mission of training professionals in Medicine, Nursing, Physiotherapy, and Psychology who not only master technical skills but also essential transversal learning outcomes. To achieve this, priority is given to the application of active learning methodologies such as Problem-Based Learning (PBL), Clinical Scenario-Based Learning (CSBL), and simulation, all of which are aligned with Ignatian Pedagogy. To support this teaching innovation, the Commission on Learning Methodologies in Health Sciences (CoMACS, from the Spanish) was established as a natural evolution of the Medical and Health Education Unit. Its objective is to coordinate the implementation of these methodologies, train faculty, and ensure educational quality. Its achievements include the full implementation of PBL in the Bachelor’s Degree in Medicine, the consolidation of CSBL in the Bachelor’s Degree in Nursing, and the development of simulation programs. This model has strengthened teaching innovation and educational excellence, positioning itself as a benchmark at the University of Deusto.

## Introduction

### Ignatian Pedagogy in Higher Education

The University of Deusto (UD), with a history spanning more than a century, is a unique benchmark in higher education, not only in Spain but also globally, intrinsically linked to the Society of Jesus. Founded in 1886, its identity is shaped by the principles and values of the Jesuit order, which sets it apart from other universities by integrating a profound humanistic and ethical dimension into its educational approach (Cava Mesa & Leonardo Aurtenetxe, 2011). Since its creation by Ignatius of Loyola in the sixteenth century, the Society of Jesus has given fundamental priority to education as a tool for the integral development of the individual and social transformation (Herrera, 2024; O'Malley, 1995). This pedagogical vocation manifested itself early on in the founding of schools and universities around the world, establishing a global educational network that has significantly influenced the history of thought and science.

The influence of the Society of Jesus on teaching is not limited to the mere transmission of knowledge, but is based on a particular educational philosophy known as Ignatian Pedagogy. This is characterized by personalized attention to students, the encouragement of critical thinking, the promotion of reflection and discernment, and the search for the “magis”—understood as excellence or “more” in all areas of life—(The International Center for Jesuit Education, n.d.). Unlike more traditional pedagogical models focused on memorization or the accumulation of data, Ignatian Pedagogy emphasizes the development of analytical, synthetic, and evaluative skills, preparing students to face complex challenges and make informed decisions in a constantly changing world (Avellán et al., 2023).

### The Ledesma–Kolvenbach Paradigm

This is embodied in the Ledesma–Kolvenbach paradigm, which refers to four relevant components of Ignatian Pedagogy. The Ledesma–Kolvenbach paradigm has its origins in the thinking of the Jesuit theologian and educator Diego de Ledesma, who in the sixteenth century gave four reasons why the Society of Jesus should take responsibility for educational institutions. These four principles were redefined in the twenty-first century by the former Superior General of the Society of Jesus, Fr. Peter Hans Kolvenbach, as the four ultimate goals of Jesuit education. In condensed form, Fr. Kolvenbach named them by their Latin names: “utilitas,” “iustitia,” “humanitas,” and “fides”: utility, justice, humanity, and faith. This paradigm helps us to better understand the mission of universities guided by the Society of Jesus, such as the University of Deusto, inspiring not only educational management, but also teaching, research, and the social impact of their actions, as well as the profile of the professionals to be trained (Coppetti, 2011). Currently, this approach is embodied in active pedagogical methodologies that promote student participation, such as Problem-Based Learning, Case Studies, and the integration of theory with practice.

### Contemporary Relevance

The implications of Ignatian Pedagogy in today’s higher education are profound and highly relevant (Durán, 2023). In a global context characterized by uncertainty, cultural diversity, and the need for professionals with a solid ethical foundation, the comprehensive education offered by Jesuit universities takes on added value. The concern for forming “men and women for and with others” (Arrupe, 1973) translates into academic programs that, in addition to disciplinary excellence, incorporate a dimension of social commitment, civic responsibility, and sensitivity to the realities of injustice and inequality. This is reflected in the promotion of service-learning projects, the inclusion of ethical reflection in the curriculum of all disciplines, and the promotion of research that contributes to the common good. In essence, the University of Deusto, as a Jesuit institution, seeks not only to equip its students with the necessary professional skills, but also to shape conscious, competent, compassionate, and committed individuals (Coppetti, 2011). In the following sections of this article, we will explore how these principles translate into the specific educational practices of the University of Deusto and their impact on the preparation of future leaders and citizens.

### Transversal Learning Outcomes at the University of Deusto

Since the Bologna Declaration in 1999, the University of Deusto, like many others, has reoriented its pedagogical approach to respond to the new demands of the knowledge era and adapt to the European Higher Education Area. With this in mind, the 2000–2001 academic year marked the beginning of a period of pedagogical renewal, moving from a model focused primarily on the teacher to one focused on student learning and skills development. Thus, together with other European universities that had similar approaches, the Tuning Project (Tuning Academy, 2000) emerged, led from the outset by the University of Deusto and the University of Groningen (Netherlands). This project has come a long way and has not only spread throughout the European university sphere, but has also reached other continents (Africa, America, Asia, etc.).

In its aim to promote autonomous and meaningful learning, it was seen that, in addition to the specific learning outcomes of each degree, graduates had to incorporate learning outcomes known as “generic or transversal” learning outcomes, such as teamwork and oral communication, among others. Thus, at the UD, from extensive lists of learning outcomes, those necessary for the training of its graduates were selected according to previously defined academic and professional profiles, thus responding to its own university mission as a project of the Society of Jesus.

**Fig. 1:**
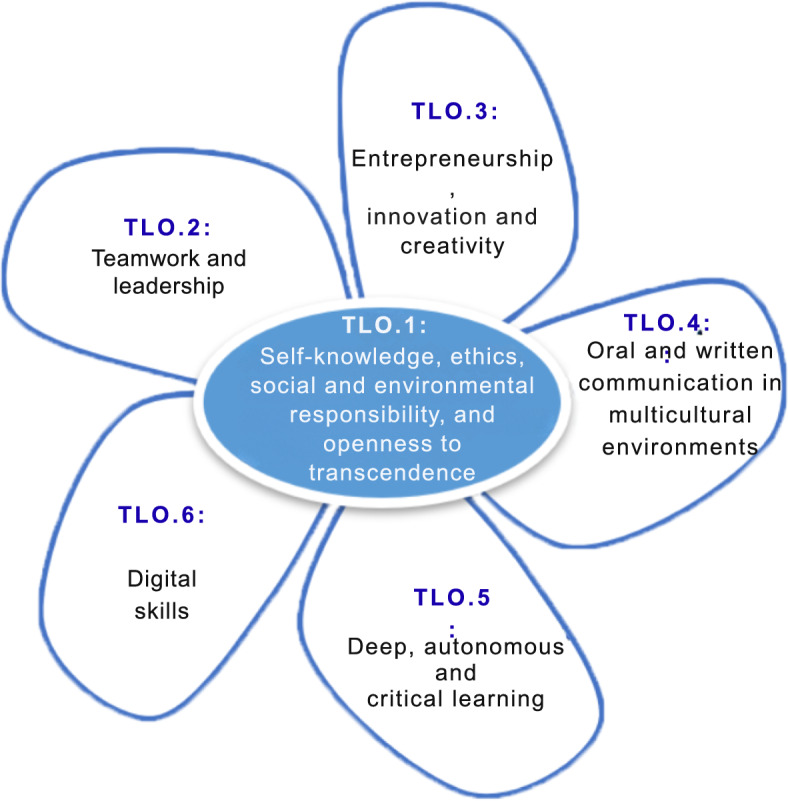
Helix as a proposal for transversal learning outcomes to be developed in the teaching unit

After fifteen years of applying the model, in September 2016, the UD began a process of reflection on the transversal learning outcomes (TLOs) to be developed, taking into account the classification of skills proposed at that time by the National Agency for Quality Assessment and Accreditation, ANECA (2015), which differentiated between four types of skills or learning outcomes: basic, transversal, general, and specific. After reviewing the educational models of various universities after 2010, documentation relating to the Society of Jesus, UNIJES, and the UD itself, the educational models of Jesuit universities, and documentation from observatories and national and international public educational institutions, the key elements that define a graduate of the University of Deusto and, in addition, their competency profile were identified. After analyzing all this documentation, six transversal learning outcomes were selected and defined, represented visually as shown in Fig. 1, with TLO.1, related to knowledge, ethics, social and environmental responsibility, and openness to transcendence, positioned as the axis of a propeller, as it is the competency that must drive and permeate the TLOs.

Below, we list the six transversal learning outcomes of the UD along with their definitions (Table 1):

**Table 1:** Transversal learning outcomes: name and definition

TLO.1: Self-knowledge, ethics, social, and environmental responsibility and openness to transcendence Acting in an ethical, egalitarian, inclusive, responsible, and sustainable manner toward oneself, others (men and women for others), society (social justice), and the planet as a whole (environment), while considering the big questions in life	
TLO.2: Teamwork and leadership Working collaboratively to achieve common goals by exchanging constructive contributions, mediating conflicts, sharing knowledge, and assuming commitments and responsibilities in the service of the team, taking on the role of team leader when the occasion or context requires it	
TLO.3: Entrepreneurship, innovation, and creativity Develop new ideas, actions, and projects that have a positive impact on the environment, turning ideas into actions, making decisions, and taking risks	
TLO.4: Oral and written communication in multicultural environments Communicate orally and in writing to interact effectively with others; listening, expressing, and conveying feelings, knowledge, ideas, and arguments clearly, rigorously, and convincingly; using a variety of expressive resources, both orally and in writing; using appropriate linguistic resources and formats and adapting to circumstances, audience types, and diverse cultural contexts, using a variety of languages. Multilingual communication also requires intercultural understanding, valuing cultural diversity, and showing interest and curiosity about languages and intercultural communication	
TLO.5: Deep, autonomous, and critical learning Updating one’s own learning, questioning habitual ways of acting, and asking questions from a critical perspective, reflecting on one’s own knowledge and way of learning	
TLO.6: Digital skills Work effectively and efficiently with technology to process information, as well as communicate critically and responsibly in the digital world	

Each of these TLOs is considered as a subject learning outcome and is therefore specified in the learning outcomes for each subject.

TLO.1 aims to foster a responsible commitment to oneself and to the social, cultural, and natural environment. It therefore includes self-awareness and the discernment necessary for making decisions that affect oneself, other people, and society.

TLO.2 refers to personal and social skills, as well as leadership.

TLO.3 encompasses creativity, innovation, risk-taking, and project management.

TLO.4 includes elements of multilingualism, effective communication, and interculturality.

TLO.5 aims to develop an interest in learning, the necessary reflection on one’s own learning, and questioning things.

Finally, TLO.6 refers to technical skills, social interaction skills, and ethical-professional skills.

As TLO.1 is the hallmark skill of the University of Deusto, its learning outcomes are also shown in Table 2.

**Table 2:** Learning outcomes for TLO.1 subjects

TLO.1: Self-knowledge, ethics, social, and environmental responsibility and openness to transcendence Act in an ethical, egalitarian, inclusive, responsible, and sustainable way, with oneself, with others (men and women for others), with society (social justice) and with the planet as a whole (environment), asking the big questions of life	
TLO.1.1: Reflect on oneself and on the meaning of life in order to make personal and professional decisions TLO.1.2: Reflect on the consequences of one’s own actions within a values-based framework TLO.1.3: Act honestly and respectfully in a variety of contexts related to academic and professional practice TLO.1.4: Apply professional ethics to resolve real or simulated cases TLO.1.5: Analyze the social and environmental impact of academic and professional actions and proposals in their area of study TLO.1.6: Carry out actions aimed at implementing environmental responsibility to protect natural resources and nature as a whole	

These guidelines set by the University must be implemented by the faculties, which are ultimately responsible for the degrees.

### Educational Principles of Faculty of Health Sciences at the University of Deusto

The Faculty of Health Sciences at the University of Deusto was founded in 2020 with a clear commitment to training healthcare professionals from a humanistic perspective, backed by a high-quality teaching staff, advanced facilities, and innovative methodologies. This new project embodies the ideal of training people in the fields of medicine, nursing, physiotherapy, and psychology to be free, reflective, solidly grounded in values, service-oriented, capable of questioning and improving the society in which they live, using up-to-date methodologies to promote meaningful learning and the desire to continue learning in the future.

One of the objectives of the Faculty of Health Sciences’ strategic plan for 2023–2026 is to strengthen the implementation of innovative teaching–learning methodologies that help develop the skills needed to respond to the present and future challenges of society, adapting to the profile of the student body.

Thus, the key methodology in the Bachelor’s Degree in Medicine at the University of Deusto is Problem-Based Learning (PBL), which promotes the comprehensive development of the individual through experiential and practical learning, a hallmark of the Society’s approach to teaching.

PBL, in line with Ignatian Pedagogy, places the student at the center of learning, encourages critical reflection, and promotes social commitment. In addition, it articulates the five elements of the Ignatian Pedagogical paradigm (context, experience, reflection, action, and evaluation) through real problems that mobilize all dimensions of being. This methodology favors Ignatian discernment, interiority, and collaborative work, guiding toward a transformative and meaningful education (Hernández Franco et al., 2022).

In line with this, the Nursing Degree has created its own active, student-centered learning methodology: Clinical Scenario-Based Learning (CSBL, ABEC by the Spanish acronym). This methodology is based on the discovery of information and the acquisition of specific and transversal learning outcomes by students through the resolution of challenges and tasks proposed by teachers, working in groups of five people guided by a tutor (Sánchez Isla et al., 2025). Students develop, above all, the phases of experimentation and reflection characteristic of Ignatian Pedagogy.

Simulation is another key active learning methodology used in the faculty. This educational tool consists of reproducing real or potential situations in a safe and controlled environment, allowing students to practice skills, solve problems, and make decisions without putting patients at risk. This tool allows learning to be progressive and adapted to the level of the students, enables the development of both specific and transversal learning outcomes, and prepares students to deal with real crisis situations (Roussin & Weinstock, 2017). In addition, it requires students to reflect deeply on what they have learned and helps in the development of experiential learning, characteristics of Ignatian Pedagogy.

Before the creation of the Faculty of Health Sciences, the University of Deusto already had a Medical and Health Education Unit (MHEU), which was part of the Teaching Innovation Unit. This unit was one of the fundamental structures for ensuring the implementation of teaching models for the different degrees offered by the Faculty of Health Sciences. Its functions included: teacher training, advising on the planning of study programs, participating in the review and improvement of subjects, and conducting research in Medical and Health Education, among others.

Medical Education Units (MEUs) at the individual level have traditionally been the driving force behind innovation and continuous improvement in the training of healthcare professionals. Medical Education Units are becoming an essential component of medical and health faculties worldwide. Although there is no consensus on the name they adopt (Medical Education Departments, Health Professions Education Departments, Medical Education Development Offices, etc.), more and more universities are recognizing the need for these units, made up of experts in health sciences education, to ensure the quality of the training for health sciences professionals at the undergraduate, graduate, and continuing education levels, as they provide the technical support and expertise needed to implement significant changes in teaching and learning processes (Aguayo-Albasini et al., 2021; Davis et al., 2005; Kerns et al., 2024).

With the creation of the new Faculty of Health Sciences, the UD Medical and Health Education Unit becomes part of the new Faculty. Given the importance and usefulness of the MHEU, in the 2024–2025 academic year it evolves into what is now known as the Health Sciences Learning Methodologies Commission (CoMACS, from Spanish).

The University of Deusto, with its deep-rooted tradition in Ignatian Pedagogy, finds in this CoMACS initiative a reflection of its educational principles. Ignatian Pedagogy, which seeks the comprehensive formation of the person, emphasizes experiential learning, critical reflection, individualized attention, and service to others. The commitment to active learning methodologies such as PBL, CSBL, and simulation aligns perfectly with this approach, as they promote student autonomy, the practical application of knowledge, and the development of a sense of social responsibility. CoMACS, therefore, not only seeks pedagogical innovation but also reinforces the institution’s identity and humanistic values.

The implementation of active learning methodologies is crucial for training healthcare professionals who not only possess solid technical skills, but also a deeply humanistic character and essential transversal learning outcomes. In an increasingly complex healthcare environment, it is vital that future professionals are able to work in teams, communicate effectively, solve problems creatively, and demonstrate empathy and ethics in their practice. Active methodologies, by focusing learning on the student and on solving real challenges, are ideal for developing these interpersonal and attitudinal skills, which go beyond purely technical knowledge.

PBL, CSBL, and Simulation all articulate the five elements of IP (context, experience, reflection, action, and evaluation) through real-life problems. They promote comprehensive development and encourage Ignatian discernment, interiority, and collaborative work. Given that the TLO.1 requires the application of professional ethics to resolve real or simulated cases and reflect on the meaning of life and the consequences of actions, problems, scenarios, or simulation cases are the main vehicle for students to face dilemmas that engage all dimensions of being. In all methodologies, students acquire specific and transversal learning outcomes through the resolution of challenges and group tasks. This is vital for TLO.1, as it develops responsibility and social commitment by facing challenges that require the application of values and interpersonal skills.

Furthermore, all of them require students to reflect deeply on what they have learned and facilitate experiential learning. They allow students to practice decision-making and managing complex situations in a safe environment. This is crucial for meeting TLO.1.4 (Applying professional ethics to resolve real or simulated cases) and TLO.1.2 (Reflecting on the consequences of one’s actions) without putting patients at risk. In essence, the commitment to these active methodologies reinforces the humanistic identity of the institution and its values, promoting student autonomy, the practical application of knowledge, and the development of a sense of social responsibility.

### Aim

The objective of this article is to describe the CoMACS as one of the principal institutional mechanisms that articulates the Ignatian Pedagogical Paradigm within Deusto’s Faculty of Health Sciences, promoting active methodologies and ensuring the development of transversal learning outcomes (TLOS).

## Methods

This article presents a descriptive institutional case study of CoMACS, based on internal documentation and records from the Faculty of Health Sciences at the University of Deusto.

With the aim of promoting teaching innovation and improving the quality of training in Health Sciences, building on the work already carried out by the university’s Medical and Health Education Unit (MHEU), the Commission on Learning Methodologies in Health Sciences (CoMACS) has been created. This commission, attached to the Vice-Dean’s Office for Academic Planning, Teaching Innovation, and Quality at the Faculty of Health Sciences of the University of Deusto, has its main objective to promote the implementation of active and innovative learning methodologies in the various degrees offered by the Faculty.

CoMACS is chaired by the Vice Dean of Academic Planning, Teaching Innovation, and Quality, who also represents the Faculty of Health Sciences on the University’s Teaching Innovation Committee. Faculty members (teaching and research staff) and technical staff from the Faculty participate in the commission. In addition, there is a liaison with the University’s Teaching Innovation Unit.

The commission is organized into three subcommissions, each focused on a specific methodology:

1. Problem-Based Learning (PBL).
2. Clinical Scenario-Based Learning (CSBL).
3. Simulation.

Each of these subcommissions is led by a faculty member with experience in the corresponding methodology, who also acts as a representative on the general commission. The rest of the team in each subcommission is made up of faculty members from the departments whose degrees apply the methodology and administrative and services staff from the technical team. All members of each subcommission have training and experience in teaching innovation to provide support to teachers.

The main objective of the CoMACS and its subcommissions is to implement active and innovative learning methodologies that foster student interest and promote autonomous, meaningful, and in-depth learning, in line with Ignatian Pedagogy.

As indicated in this document, this educational philosophy, a hallmark of the University of Deusto, goes beyond the mere transmission of knowledge. It focuses on the comprehensive development of students, working on transversal learning outcomes such as critical thinking, teamwork, and communication, with the aim of pursuing excellence (*magis*). In this way, methodologies such as Problem-Based Learning, clinical case studies, and the integration of theory and practice not only encourage active participation but also prepare future professionals to face complex challenges and make informed decisions.

Its specific objectives include promoting the adoption of active methodologies such as PBL, CSBL, and simulation, facilitating teacher training in these methodologies, supporting teachers in their implementation in the classroom, and ensuring the quality of implementation and the achievement of learning outcomes. In essence, the commission seeks to contribute to the training of professionals who are not only competent but also conscious, compassionate, and committed to society.

For their part, the subcommissions will have the specific objectives of defining criteria and standards for each methodology, training teachers in their effective implementation, coordinating with the general commission and the technical unit, and monitoring the application of the methodologies in the different degrees.

As the article reports aggregated institutional data and does not involve human participants or identifiable personal data, formal ethics committee approval was not required.

## Results

Since the creation of the MHEU, predecessor of the CoMACS, and up to the present, the following results have been achieved:

The information presented here has been drawn from CoMACS meeting minutes, institutional reports, and administrative records of training activities and teaching innovation projects from 2020 to 2025.

The first five years of the Bachelor’s Degree in Medicine have been implemented using PBL as the key methodology, and in the 2025–2026 academic year, we are in the process of implementing the sixth and final year. To make this happen, a training program has been designed, implemented, evaluated, and improved for teachers of the Bachelor’s Degree in Medicine with the aim of training them in the acquisition of teaching skills and transversal learning outcomes, in the UD training model, which closely follows the guidelines of Ignatian Pedagogy, and in different active methodologies. From 2020 to August 2025, more than 250 teachers have completed this training program.

The experience gained in recent years in PBL training has enabled us to develop a synchronous online course for teachers at universities in the UNIJES network, taught in 2024 and 2025 by members of CoMACS, in which a total of 23 people have been trained. These training courses have also been developed in face-to-face format at some Spanish universities.

In addition, from the outset, there has been a commitment to disseminating the work carried out and the results obtained in Teaching Innovation projects, at Good Practice Conferences, congresses, and in scientific publications (Gutierrez-Aguirregabiria et al., 2024, 2025a, 2025b, 2025c; Romero-Yesa et al., 2024; Sáenz Bilbao et al., 2022, 2023; Velasco del Castillo et al., 2025).

The CSBL methodology has been specifically designed at the University of Deusto for its Nursing Degree, where it has been fully implemented. Its characteristics, steps, and how it is integrated into a university degree are described in a book-format publication that facilitates its adoption in those Nursing Degrees that wish to do so (Sánchez Isla et al., 2025).

In addition, the CSBL subcommission provides support to teaching staff and ensures compliance with the methodological objectives in the classes taught.

Furthermore, the simulation subcommission has worked to reach a consensus on what simulation methodology entails and has compiled the different simulation exercises carried out in the various degrees offered by the faculty in order to achieve a common approach to simulation. In addition, two editions of a 20-h simulation course have been organized, taught by external experts, which has been attended by 40 teachers from the two campuses of the University of Deusto.

Currently, the possibility of implementing and expanding this methodology to other degrees in the Faculty of Health Sciences that do not yet use it regularly, such as the degree in Psychology, is being explored. The aim is to replicate the success of clinical simulation in training key skills, such as decision-making, communication with patients, and managing complex situations, adapting them to the specific contexts of each discipline.

There are also plans to extend this practice to other faculties of the university, outside the healthcare context, recognizing that simulation is a cross-cutting pedagogical tool that can enrich the training of students in areas as diverse as law, economics, and education, offering practical scenarios for the development of interpersonal skills and problem-solving in a safe and controlled environment. This joint effort seeks not only to unify methodologies, but also to foster a culture of practical and experiential learning at the institutional level.

## Limitations

Incorporating a commission on learning methodologies into a faculty requires the support from the faculty itself, allowing it to allocate human resources to the creation of this working group, which in turn requires a conviction that these working groups bring benefits to the faculty. In addition, there must be a number of faculty members trained in teaching innovation who are interested in this area and willing to devote part of their working hours to the commission’s tasks.

A key limitation of this article is the current scarcity of formal outcome data directly demonstrating the long-term impact of CoMACS. This is primarily due to the fact that the Commission has only been fully operational for a short period (one academic year).

## Discussion

Medical Education Units are becoming increasingly common in faculties offering health degrees, and the UD has been committed to having a MHEU since the design of the Bachelor’s Degree in Medicine began, even before the creation of the Faculty of Health Sciences. Committed to higher education in which students play a leading role and giving transversal learning outcomes the importance they deserve, always following Ignatian Pedagogy, the UD has committed to the evolution of the MHEU into a commission on learning methodologies in health sciences (CoMACS) in order to encourage the implementation of learning methodologies and promote the training of faculty staff. Having a team of experts in Teaching Innovation dedicated to CoMACS has made it easier to improve the training offered to faculty members, implement different learning methodologies in the faculty’s degree programs, and motivate faculty to convey to students the importance of transversal learning outcomes and implement the concepts of Ignatian Pedagogy.

CoMACS’ plans to improve scenario writing (and problems and cases, according to the methodology) and the creation of good practice guides are essential to ensure the uniformity and effectiveness of the implementation of complex scenarios, such as those that mobilizes the Transversal Learning Outcome 1 (TLO.1) throughout the Faculty. This is achieved through standardization, specialized training and quality assurance. The following objectives are therefore proposed:

1. Improving scenario writing in the three methodologies:
  CoMACS plans to design a training program to improve the writing of scenarios/problems/cases. Given that each degree has key methodologies and these are the vehicle for mobilizing Ignatian Pedagogy (IP), improving their quality ensures that these scenarios/problems/cases remain real situations that mobilize all dimensions of being.
  Improving the writing ensures them to be complex enough to compel students to apply professional ethics (TLO.1.4) and reflect on themselves and the meaning of life (TLO.1.1), ensuring that these transversal learning outcomes are addressed effectively and consistently across all subjects.
2. Creation of Good Practice and Reference Guides:
  CoMACS aims to write a good practice guide for PBL and Simulation, similar to the one already published for CSBL.
  These guides are intended to define criteria and standards for each methodology. Standardization is crucial for uniform implementation, ensuring that all teachers trained by CoMACS apply the methodologies consistently to achieve learning objectives.
  Specifically for Simulation, CoMACS will also produce quick reference guides on simulation to support teachers who wish to incorporate this methodology into their classes, which is fundamental to the development of experiential learning and the deep reflection required by IP and TLO.1.
3. General Teacher Training for Effectiveness:
  In addition to specific plans, CoMACS will design a training plan that includes courses on how to give feedback or how to evaluate students effectively. These are teaching skills necessary in all active methodologies. This general training ensures the effectiveness of the tutor in the reflection stage, which is where Ignatian discernment is consolidated and ethical and attitudinal competencies are evaluated (TLO.1).

In summary, CoMACS seeks to ensure the quality of implementation and the achievement of learning outcomes by defining standards (guidelines) and providing ongoing training for teachers to handle complex scenarios in a consistent manner.

Furthermore, the success of the Ledesma–Kolvenbach paradigm in the institutional management of the University of Deusto (UD) is demonstrated by the replication of the CoMACS model by other faculties, such as Engineering, which has created its own commission, the CoMAI (Commission for Learning Methodologies in Engineering).

This replication proves the institutional commitment to quality and continuous improvement:

1. Institutional Validation of the Model: the Ledesma–Kolvenbach paradigm inspires educational management, teaching, research, and the social impact of the university’s actions. The CoMACS model reflects these principles, as it seeks comprehensive training and aims to ensure the quality of implementation and the achievement of learning outcomes.
2. Replication of Quality Structures: new commissions such as the one created in the Faculty of Engineering have similar functions to those of CoMACS, although adapted to their specific characteristics, demonstrating that the success of CoMACS has made it a commission with a long future ahead and a benchmark at the UD.
3. Commitment to Continuous Improvement: The evolution of the Medical Education Unit (MHEU) to CoMACS demonstrates the UD’s commitment to fostering the implementation of learning methodologies and promoting teacher training. The adoption of similar structures in other faculties indicates that the UD is investing at an institutional level in the technical support and expertise necessary to implement significant changes in teaching and learning processes, ensuring that education remains focused on the student and the development of transversal learning outcomes.
